# Intracellular Survival of *Staphylococcus aureus* in Endothelial Cells: A Matter of Growth or Persistence

**DOI:** 10.3389/fmicb.2017.01354

**Published:** 2017-07-19

**Authors:** Guillaume Rollin, Xin Tan, Fabiola Tros, Marion Dupuis, Xavier Nassif, Alain Charbit, Mathieu Coureuil

**Affiliations:** ^1^Université Paris Descartes, Sorbonne Paris Cité, Bâtiment Leriche Paris, France; ^2^Institut National de la Santé et de la Recherche Médicale U1151 - Centre National de la Recherche Scientifique UMR 8253, Institut Necker-Enfants Malades, Equipe 11: Pathogénie des Infections Systémiques Paris, France; ^3^Assistance Publique – Hôpitaux de Paris, Hôpital Necker Enfants Malades Paris, France

**Keywords:** *Staphylococcus aureus*, USA300, intracellular persistence, endothelial cells, SCV

## Abstract

The Gram-positive human pathogen *Staphylococcus aureus* is a leading cause of severe bacterial infections. Recent studies have shown that various cell types could readily internalize *S. aureus* and infected cells have been proposed to serve as vehicle for the systemic dissemination of the pathogen. Here we focused on the intracellular behavior of the Community-Associated Methicillin-Resistant *S. aureus* strain USA300. Supporting earlier observations, we found that wild-type *S. aureus* strain USA300 persisted for longer period within endothelial cells than within macrophages and that a mutant displaying the small colony variant phenotype (Δ*hemDBL*) had increased intracellular persistence. Time-lapse microscopy revealed that initial persistence of wild-type bacteria in endothelial cells corresponded to distinct single cell events, ranging from active intracellular bacterial proliferation, leading to cell lysis, to non-replicating bacterial persistence even 1 week after infection. In sharp contrast, Δ*hemDBL* mutant bacteria were essentially non-replicating up to 10 days after infection. These findings suggest that internalization of *S. aureus* in endothelial cells triggers its persistence and support the notion that endothelial cells might constitute an intracellular persistence niche responsible for reported relapse of infection after antibiotic therapy.

## Introduction

The gram-positive bacterial pathogen, *Staphylococcus aureus*, can colonize multiple anatomic sites within its human host, including nares, respiratory tract and skin. Under defined conditions, *S. aureus* is also capable of systemic dissemination and can cause skin and soft tissue infections, pneumonia as well as sepsis, endocarditis, bones and joints infections (Thomer et al., 2016). The rates of infections caused by staphylococci, both community- and hospital-acquired strains, are regularly escalating (Laupland and Church, 2014). However, treatment of these infections is becoming increasingly difficult due to the prevalence of multidrug-resistant strains. In particular, *S. aureus* USA300, an epidemic community-associated methicillin-resistant strain (CA-MRSA), has now emerged as the predominant cause of methicillin-resistant *S. aureus* (MRSA) infections in the United States and is continuously spreading around the world (DeLeo et al., 2010).Initially classified as strict extracellular pathogen, *S. aureus* is now considered as a non-classical facultative intracellular pathogen (Sendi and Proctor, 2009). Indeed, numerous cell types can ingest *S. aureus* and the bacterium is able to persist within these cells for quite variable periods of time (Fraunholz and Sinha, 2012; Strobel et al., 2016).

Relapse of *S. aureus* infection after a well-conducted antibiotic treatment constitutes a major health issue. One hypothesis is that relapse may result from a lack of access of the antibiotic to the site of infection, especially to the intracellular niche. The molecular mechanisms underlying *S. aureus* virulence have been extensively studied and have been shown to be mediated by a multitude of virulence attributes, including adhesins and toxins that are regulated by complex networks of regulatory systems (Somerville and Proctor, 2009; Felden et al., 2011; Ibarra et al., 2013; Foster et al., 2014). Important contributions have been made over the past 10 years regarding the physiological and metabolic status of intracellular *S. aureus* (Sendi and Proctor, 2009; Tuchscherr et al., 2011; Proctor et al., 2014; Thammavongsa et al., 2015). However, the time course of *S. aureus* intracellular persistence is still poorly characterized.

The aim of the present work was to better characterize the dynamic stability of intracellular persistence of the CA-MRSA strain *S. aureus* USA300-LAC. We focused on endothelial cells that have been shown to readily internalize staphylococci *in vitro* (Strobel et al., 2016). We quantitatively and qualitatively followed the behavior of intracellular bacteria, by using a combination of confocal and electron microscopy and live-cell imaging. Our results highlight the heterogeneity of *S. aureus* behavior during cell infection and suggest that intracellular survival is a selective pressure that selects transient slow growing bacteria that are able to persist inside cell cytosol for several days and evade the cells by a mechanism yet to be determined.

## Materials and methods

### Strains and culture conditions

The epidemic clone *S. aureus* USA300-LAC (designated USA300-WT) was provided by the Biodefense and Emerging Infections Research Resources (BEI). The GFP-expressing strain (designated USA300-GFP) was generated by curing the p03 plasmid from USA300-WT and introducing the pCN57-GFP recombinant plasmid (obtained from the BEI) by electroporation (electroporator settings: 2,450 V, 100 Ω, 25 μF, time constant = 2.3–2.5 ms). Growth curve were performed in BHI broth. Internalization rate and survival inside the EA.hy926 endothelial cell line were similar between the GFP strain and the parental strain.

### Construction of the *S. aureus* Δ*hemDBL* triple deletion mutant

We simultaneously inactivated the three consecutive genes *hemD hemB and hemL* of the heme biosynthesis locus in wild-type *S. aureus* USA300 strain and substituted them by the kanamycine resistance gene *npt*II fused with pG*ro* promoter. For this, we used the pMAD-temperature-sensitive shuttle vector system (Arnaud et al., 2004). Briefly, the recombinant plasmid pMAD-Δ*hemDBL* was constructed by overlap PCR. First, the two regions (upstream 690 bp, downstream 458 bp) flanking *hemB* and the *npt*II gene (fused with pG*ro* promoter 1,091 bp) were amplified by PCR using the following pairs of primers: i) Primers p1 and p2 amplified the region upstream (hemBUp) of the start codon of the *hemB* coding sequence (p1 5′-CGGAATTCCCGGTTGAGTTAGGCAAAACAGTGAG-3′ and p2 5′-TTTAGCTCGACTAATCCATACAAGGTCCGTGCTGTTTGTTCTCC-3′); primers p3 and p4 amplified the region downstream (hemBDown) of the *hemB* stop codon (p3 5′-CCTTCTTGACGAGTTCTTCTGAGCCTGGTGGTGTAAATAGTCCAG-3′ and P4 5′-CGGGATCCCACCAGGAGAATCCGGCAATCC-3′); iii) primers p5 and p6 amplified the *npt*II gene (p5 5′-TTGTATGGATTAGTCGAGCTAAA-3′ and p6 5′-TCAGAAGAACTCGTCAAGAAGG-3′).

The region hemBUp-npt-hemBDown (2,239 bp) was then amplified by two-step overlap PCR. A first overlap PCR was realized to amplify the region hemBUp-npt, using the hemBUp and npt PCR fragments. The amplified product was cloned in pMiniT 2.0 (using the PCR Cloning Kit New England BioLabs). A second overlap PCR was then realized for the amplification of region hemBUp-npt-hemBDown, using hemBUp-npt and hemBDown PCR fragments. The resulting PCR product was cloned in pMiniT 2.0 (to yield recombinant plasmid pMiniT/hemBUp-npt-hemBDown). This recombinant plasmid was digested with *Bam*HI and *Eco*RI (New England BioLabs) and the *Bam*HI-*Eco*RI hemBUp-npt-hemBDown DNA fragment was finally subcloned into *Bam*HI-*Eco*RI-digested pMAD. All PCR reactions were realized using Phusion High-Fidelity DNA Polymerase (ThermoScientific) and PCR products were purified using QIAquick PCR purification kit (Qiagen).

The pMAD-Δ*hemDBL* plasmid was first introduced into *E. coli* DH5α and then transferred to *S. aureus* RN4220 prior to electroporation into *S. aureus* USA300. A standard two-step allelic exchange procedure was used (Arnaud et al., 2004) to create the chromosomal *S. aureus* USA300Δ*hemDBL* mutant. The Δ*hemDBL* mutant strain was finally checked for loss of the corresponding wild-type genes by PCR sequencing (GATC Biotech) using specific primers (p7 5′-CGGAATTCGTCGGAGGCAAAGGCTTATTTG-3′ and p8 5′-CCGACTCTGAAACCAGTCATTACTTC-3′).

### Cell lines

To study *S. aureus* interaction with endothelial cells we used the EA.hy926 cell line (ATCC® CRL-2922™), originally derived from human umbilical vein. EA.hy926 cells were grown in Dulbecco's modified Eagle high glucose medium (DMEM, Dominique Dutscher™) supplemented with 10% fetal bovine serum (FBS, Eurobio™). Culture media contained 1% penicillin/streptomycin and 1% amphotericin B. Antibiotics were removed from culture medium prior to infection.

The human monocytic cell line THP-1 (ATCC® TIB-202™) was grown in Roswell Park Memorial Institute medium (RPMI-1640) supplemented with 5% FBS. Two days before infection, phorbol myristate acetate 200 ng mL^−1^ (PMA) was added to the culture medium to induce monocytes differentiation into macrophages.

All cells were incubated in a humidified 5% CO2 atmosphere at 37°C.

### *In vitro* infection

Four days prior to infection cells were seeded in plastic 12-well plates in culture medium without antibiotic. At time of infection each well contained a monolayer of ~5 × 10^5^ cells. Overnight cultures of *S. aureus* in BHI were diluted in BHI broth to an optical density 600 nm (OD_600_) of 0.05. Bacteria were grown at 37°C until the culture reached the initial-middle log phase (OD_600_ = 0.6), and then diluted in 1mL infection medium. Bacteria were added at a multiplicity of infection (MOI) of 1 and placed in a humidified 5% CO2 atmosphere at 37°C for 1 h.

One hour after infection, each well was washed three times with 1 mL of phosphate buffer saline (PBS) containing 300 μg.mL^−1^ gentamicin, to remove extracellular bacteria. Cells were then incubated with 1 mL of cell culture medium containing 50 μg.mL^−1^ gentamicin. Infected cells were kept in a humidified 5% CO2 atmosphere at 37°C. Importantly, gentamicin is an antibiotic with a high bactericidal effect on *S. aureus* USA300 (CMI = 2 μg.mL^−1^) and a very poor penetration inside eukaryotic cells.

At chosen times, the culture medium containing gentamicin was removed from infected cells and wells were washed three times with antibiotic free PBS. Cells were lysed with 1mL of distilled water during 15 minutes (min) and mechanically detached and serial dilutions were plated on Trypticase Soy Agar plates. Colony forming units were numerated after 24 h (h) at 37°C.

#### Cytochalasin D assay

We used Cytochalasin D, a fungal metabolite that binds to actin and reversibly modifies its polymerization, to inhibit *S. aureus* internalization into EA.hy926 endothelial cells. One hour before infection, cytochalasin D (Sigma) 50 μg.mL^−1^ was added to cell culture medium and maintained during the first hour of infection.

#### Re-seeding of *S. aureus*

EA.hy926 were infected as described above. At day 7 and 10 after infection, extracellular and intracellular bacterial load was assessed (**Figure 2A**). Cell monolayers were washed 3 times with PBS containing gentamicin 300 μg.mL^−1^ and 3 times with antibiotic free PBS. After 8 or 24 h of incubation in antibiotic-free medium the supernatant was collected, centrifugated and the pellet was resuspended in PBS before being plated on TSA for bacterial count. Then, cells were lysed and plated on TSA for intracellular bacteria count. Cells that were infected for 24 h were washed after 8 h and then incubated 16 additional hours in antibiotic-free medium.

### Microscopy

#### Immunofluorescence microscopy

EA.hy926 cells were grown to confluence on 12-mm diameter glass coverslips coated with 5 μg per cm^2^ of rat tail collagen type I. Immunofluorescence assays were performed 1 h post infection by USA300-GFP and the day of experiment. Cells were fixed in PFA 4% in PBS for 20 min and permeabilized for 5 min with PBS containing 0.1% Triton X-100. Cells were incubated with DAPI 1 μg.mL^−1^ and Alexa-633 conjugated phalloidin 1:50 in PBS containing 1% bovine serum albumin (BSA) for 1 h. After several washes, coverslips were mounted in mowiol. Image acquisition was performed on confocal microscope (Leica™ TCS SP5). Images were collected and processed using Image J software. Each image shown corresponds to the Z-projected maximal intensity signal for each fluorochrome.

#### Time lapse microscopy

EA.hy926 cells were grown to confluence on Ibidi™ μslide 8-wells and infected with USA300-GFP at a MOI of 1 as described above. Cells were incubated in cell culture medium containing 2.5 μg.mL^−1^ propidium iodide and 50 μg.mL^−1^ gentamicin. Image acquisition of infected cells was performed with an Apotome CO2 microscope (Zeiss™). One image was acquired every 20 min during 64 h. Images were collected and films were processed using Image J software. Each image corresponds to the Z-projected maximal intensity signal for GFP and to the projected median intensity for bright light.

The mutant strain USA300Δ*hemDBL* was imaged using the IncuCyte™ technology. EA.hy926 cells were grown to confluence in ImageLock 96-well plates (Essen BioScience Inc. Ann Arbor, MI, USA) and infected with the mutant strain USA300Δ *hemDBL*-GFP (at a MOI of 1). Cytotox Red reagent (Essen Bioscience) was added to the medium (final concentration of 125 nM). Plates were incubated and monitored at 37°C for 6 days in an IncuCyte™ (Essen BioScience), an incubator equipped with a fully automated phase contrast microscope. Images were taken every 20 min. The microscope had a 20 X objective. Time-lapse video were generated by using Image J software.

#### Transmission electron microscopy

EA.hy926 were grown and infected with USA300-WT as previously described. One hour after infection and at day 3 and 7 infected cells were fixed with glutaraldehyde 3% in phosphate buffer during 1 h. Cells were then post-fixed with osmium tetroxide to stabilize lipids and enhance contrast. Sample were then dehydrated with sequential ethanol baths (from 25 to 100%) and embedded in Epon 812 resin with a 48 h polymerization time at 60°C. Embedded samples were sliced in 90 nm thick pieces with an ultramicrotome and laid down on a copper observation grid. Image acquisitions were performed with a JEOL 1011 transmission electron microscope (tungsten filament) at the Institut Cochin (Paris, France).

### Statistics

*In vitro* experiments were at least repeated twice in triplicates. Data were analyzed using GraphPad Prism software. Statistical significance was assessed using Student *t*-test. In figures all results correspond to mean ± SEM.

## Results

### Persistence of intracellular *S. aureus In vitro*

Whereas several earlier studies have described the capacity of *S. aureus* to persist inside human cells, including non-professional phagocytes such as endothelial cells, other studies clearly showed the extreme propensity of this pathogen to rapidly kill infected cells (Loffler et al., 2014; Jubrail et al., 2016; Strobel et al., 2016). These apparent discrepancies are likely due the heterogeneity of protocols used to infect cells. Moreover, the vast majority of the studies related to *S. aureus* intracellular persistence have focused on early time points (generally up to 24 h post infection).

In this work, our aim was to follow the behavior of *S. aureus* USA300-LAC (referred as to USA300 in this work) when internalized into endothelial cells and to understand how this fast growing extracellular pathogen may survive in the cytosol of these cells. We decided to follow the outcome of intracellular *S. aureus* internalized during the first hours of the infection. Therefore, we adapted a gentamicin protection assay as follows: (i) after 1 h, infected cells were first washed three times with a solution containing 300 μg.mL^−1^ gentamicin (to eliminate all extracellular bacteria); and (ii) the experiments were pursued in the constant presence of 50 μg.mL^−1^ gentamicin in the culture media, to ensure elimination of extracellular staphylococci continuously escaping from lysed infected cells. The antibiotic gentamicin cannot penetrate into eukaryotic cells and has been shown to be poorly effective against intracellular *S. aureus* (Imbuluzqueta et al., 2012; Mohamed et al., 2014).

**Figure 1:**
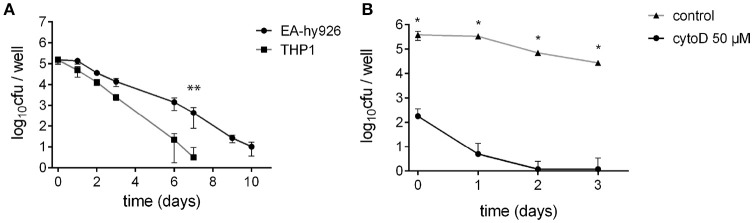
*S. aureus* can invade and survive inside various cell types. **(A)** *S. aureus* survival inside non-professional-phagocyte cells: EA.hy926 endothelial cells derived from human umbilical vein and THP-1 monocyte-macrophages. ^**^Wilcoxon-Mann-Whitney *U*-test (p < 0.05). **(B)** Endothelial cells EA.hy926 treatment with 50 μM cytochalasin D significantly reduced *S. aureus* uptake. ^*^Student *t*-test (*p* < 0.01).

We first compared the ability of *S. aureus* USA300 to survive inside the Ea.Hy296 endothelial cell line and THP-1 monocytic cell line, for 10 days following infection at a multiplicity of infection (MOI) of 1 (Figure 1A). The number of viable intracellular bacteria (i.e., able to form colonies on solid medium) recovered in human endothelial cell lines remained stable during the first 24 h after the infection. Then, bacterial intracellular persistence started to progressively decrease from day 2 but was still recorded up to 10 days after infection. No viable (i.e., culturable) bacteria were recovered after 14 days. In contrast, THP-1 macrophages had almost completely eliminated bacteria at day 7.

To confirm that surviving *S. aureus* were indeed a consequence of bacterial internalization inside endothelial cells in our assay, we inhibited bacterial uptake at day 0, using cytochalasin D (Figure 1B). A severe 1,000-fold decrease in bacterial uptake was observed in EA.hy926 cells pretreated with cytochalasin D compared to that of untreated cells. As a result, no bacterium was recovered from treated cells 2 days after infection. We next focused on the characterization of *S. aureus* intracellular persistence, using endothelial EA.hy926 cells as model.

### Interaction between *S. aureus* and infected host cells

**Figure 2:**
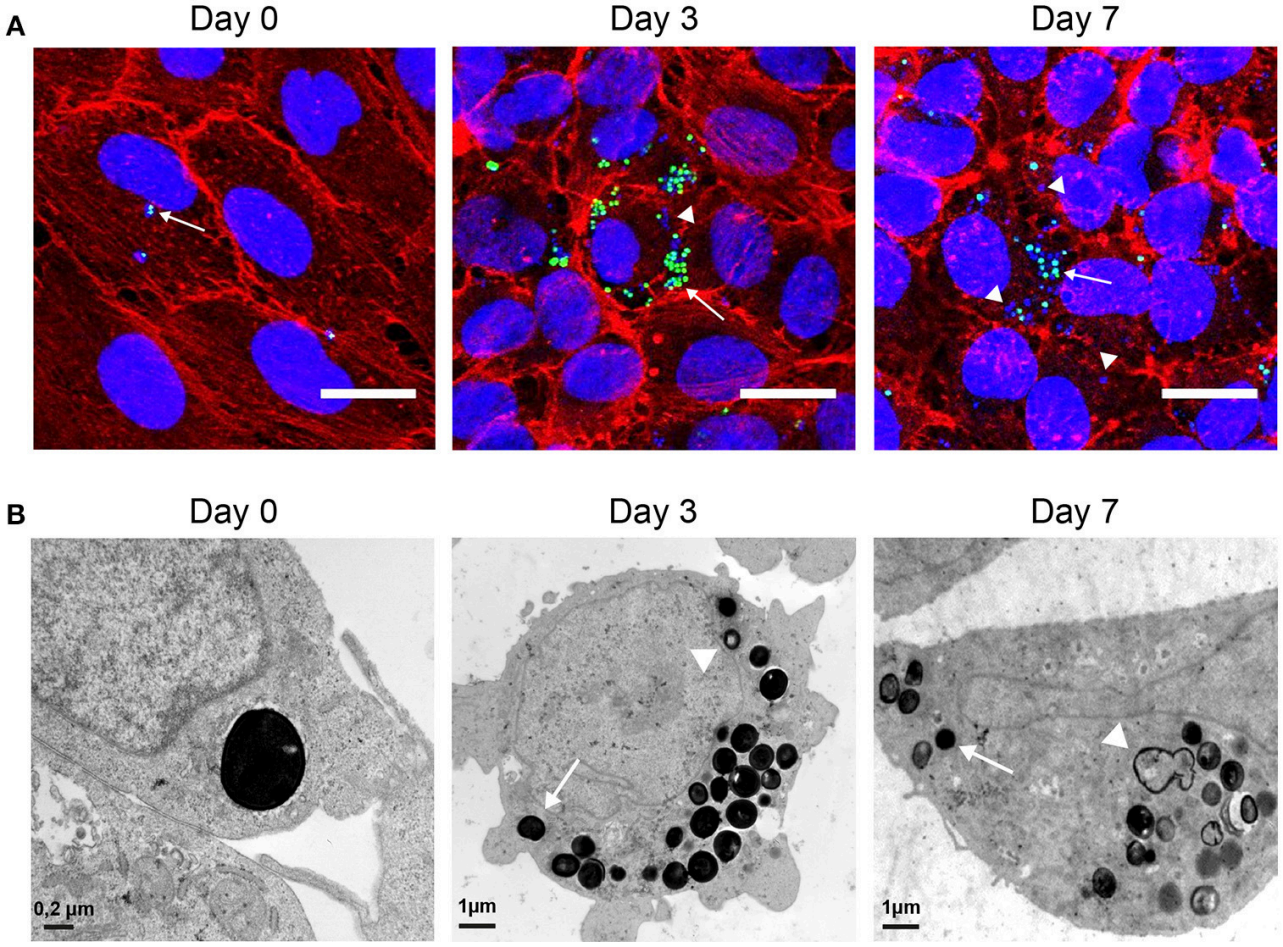
Observation of intracellular *S. aureus*. **(A)** EA.hy926 cells infected with USA300-GFP (green) observed in confocal microscopy (Z-project of max intensity) 1 h after infection and at day 3 and 7 after infection (DAPI: blue; phalloidin: red; USA300-GFP: green; scale bar = 20 μm). **(B)** EA.hy926 cells infected with USA300-WT observed in TEM 1 h after infection and at day 3 and 7. Arrows indicate living cells. Arrow heads indicate dying cells.

We next performed confocal and transmission electron microscopy (TEM) assays (Figure 2). Confocal microscopy analysis (Figure 2A) indicated that intracellular *S. aureus* actively replicate inside endothelial cells during the first 3 days of infection. After day 3, a progressive reduction in the number of GFP-expressing intracellular bacteria was observed concomitant with an increase in the number of bacteria only stained by DAPI that are likely corresponding to dead bacteria. To ascertain the fate of bacteria and that of their intracellular location inside endothelial cell we used TEM (Figure 2B). Image analysis confirmed the intracellular location of *S. aureus*. At day 0, a majority of intracellular bacteria were alive, according to their high cytoplasmic density and envelope integrity. After 3 days of infection, most of the intracellular bacteria appeared to be seriously damaged; only 23% of intracellular bacteria were still morphologically intact. As previously described, less than 5% of intracellular bacteria were surrounded by a visible vacuolar membrane (Grosz et al., 2014). After 7 days, only few intracellular bacteria were morphologically living. Confocal microscopy is consistent with this observation since dead bacteria were stained with DAPI but no longer express GFP.

### *S. aureus* USA300 uses multiple intracellular survival strategies

**Figure 3:**
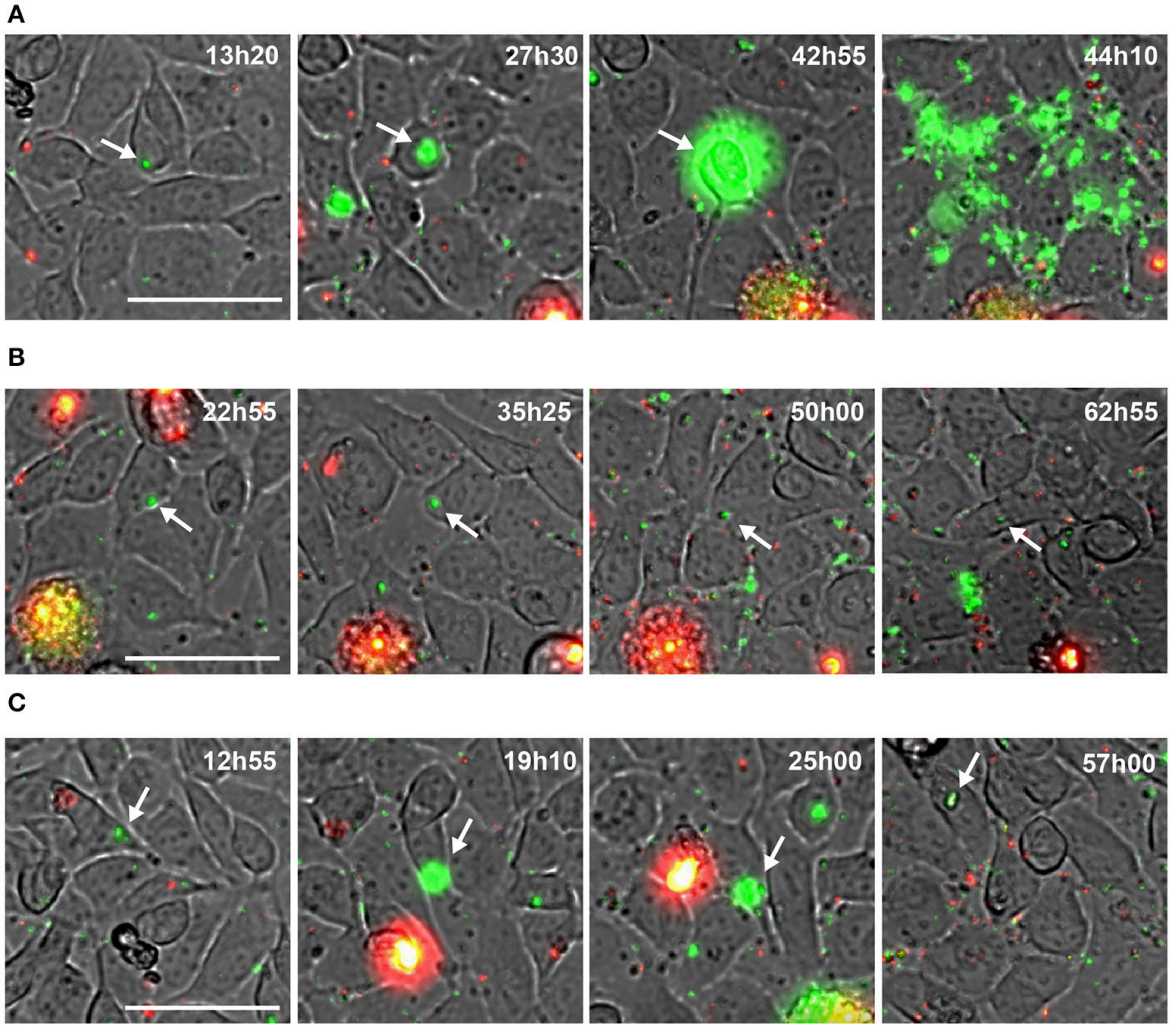
Behavior of intracellular wild type *S. aureus*. **(A–C)** Screenshots of time lapse microscopy (see Video S1–S4). EA-hy296 cells were infected by USA300-GFP and time lapse microscopy was processed for 64 h. Images were extracted at different time point. USA300-GFP: green; DNA of dying cells (propidium iodide): red. Time is in hours and minutes. Bar = 50 μm. White arrow heads point to multiplying USA300-GFP.

To follow the fate of single *S. aureus* USA300 after entry into endothelial cells, we next used time lapse-microscopy on EA.hy926 cells infected by USA300-GFP. As above, gentamicin was present throughout the experiment to prevent extracellular bacterial growth and new infection. One image was captured every 20 min during 64 h, starting 1 h after infection (Videos S1; Supplementary Materials). At the single cell level, three distinct types of behaviors were observed: (i) in the majority of cases, *S. aureus* actively proliferated during the first 48 h, leading to host cell death and to bacterial release in the extracellular medium (as illustrated in the screen captures of Figure 3A, Videos S2, S3); (ii) in some cases, however, *S. aureus* remained inside the host cell without replicating throughout the observation period Figure 3B; or (iii) after a transient phase of bacterial multiplication, host cell could control and partly eliminate intracellular *S. aureus* (Figure 3C, Video S4). Interestingly, at day 7 we did not detect intracellular replication anymore (Video S6).

Altogether these results indicate that the infection of endothelial cells by *S. aureus* is not as straightforward as suggested by colony forming units counts (CFU). In some circumstances *S. aureus* can proliferate in the cytosol of endothelial cells, leading to cell lysis, while endothelial cells may also limit bacteria proliferation leading to the emergence of persistent bacteria.

### Inactivation of the heme biosynthetic pathway improves intracellular persistence

*S. aureus* small colony variants (SCVs) constitute a slow growing subpopulation which can be recovered from patients who present persisting or relapsing infections, as it may be observed in cystic fibrosis (Goerke and Wolz, 2010). SCV phenotype (colony that exhibits significantly reduced size) can be either transient (i.e., reversible), mostly due to transcription regulation mechanisms, or permanent due to a variety of genetic defects, such as those leading to an impaired electron transport chain (due to mutations inhibiting heme or menadione biosynthesis) and/or resulting in thymidine auxotrophy (Loffler et al., 2014). Laboratory engineered mutants that mimic the SCV phenotype have been largely used to study *S. aureus* persistence and notably Δ*hemB* mutants (inactivated heme biosynthetic pathway). For example, early work of von Eiff et al. (1997) have shown that a Δ*hemB* mutant of *S. aureus* (NCTC 8325 strain) was able to persist longer than wild-type strain (over a 48 h period) within cultured bovine aortic endothelial cells, likely due to reduced cytotoxicity. More recently, one of the rare studies that addressed long term *S. aureus* persistence (Tuchscherr et al., 2011), reported that staphylococcal strains could survive within cultured host cells for up to 4 weeks and suggested that SCV formation was directly linked to chronic infection.

**Figure 4:**
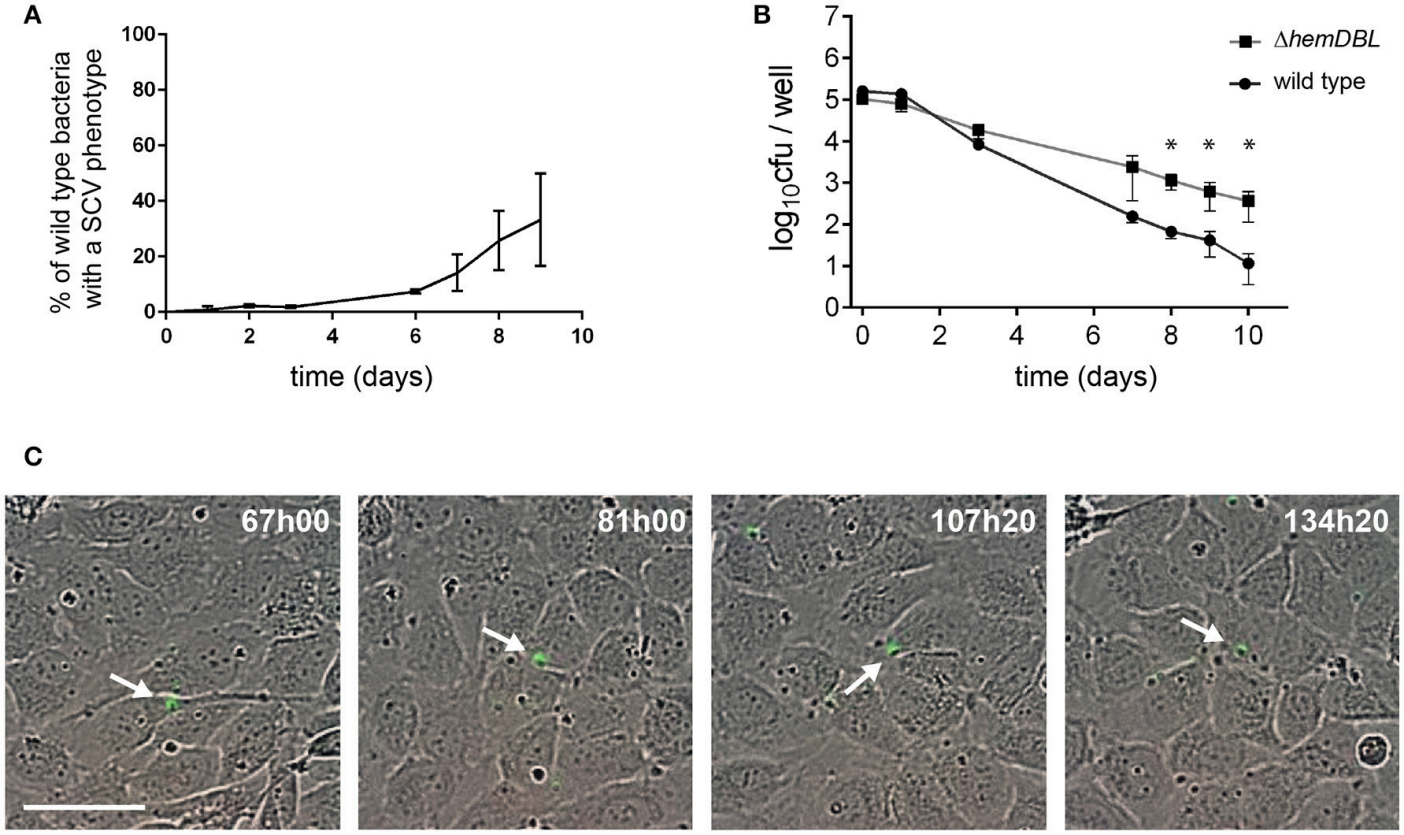
Persistence of *S. aureus* wild type and Δ*hemDBL* mutant in endothelial cells. **(A)** Percent of SCVs of wild type *S. aureus* USA300-GFP recovered from endothelial cells at different day post infection. **(B)** USA300 *S. aureus* (dot) and Δ*hemDBL* derivative (square) survival inside EA.hy926 endothelial cells. **(C)** Screenshots of time lapse microscopy (see Video S5). EA-hy296 cells were infected by Δ*hemDBL* USA300-GFP and time lapse microscopy was processed for 8 days. Images were extracted at different time point. Δ*hemDBL* USA300-GFP: green; DNA of dying cells (cytotox red reagent): red. Time is in hours and minutes. Bar = 50μm. ^*^Student *t*-test (*p* < 0.01). White arrow heads point to non-growing Δ*hemDBL* mutant bacteria.

These observations prompted us to count the proportion of SCVs in wild type *S. aureus* that infected endothelial cells (Figure 4A). Ten days after infection, only 30–40% of CFUs obtained after cell lysis showed a SCV phenotype suggesting that surviving internalized *S. aureus* only transiently slowdown their metabolism and growth. Since SCVs are known to better survive in human cells we decided to follow the fate of internalized SCVs. We thus engineered a SCV mutant by inactivating the heme utilization locus (Δ*hemDBL*) in wild-type *S. aureus* USA300 and USA300-GFP. Then we infected endothelial cells and recovered viable CFUs in infected cells (Figure 4B). While the CFUs count in Figure 4B were similar between wild type and Δ*hemDBL* mutant strains up to day 3, the Δ*hemDBL* mutant persisted more than the wild type parental strain at days 7 and 10. We then imaged the growth of our SCV mutant over a 7 day-course, using time lapse-microscopy (Figure 4C, Video S5). Neither proliferation inside endothelial cells nor cell detachment was noticed during the time of infection. This is consistent with previous reports, which indicate that SCVs induce less cell death after invasion of human cells (Tuchscherr et al., 2010, 2011). Interestingly this result recapitulated what was observed with wild type strain USA300-GFP between day 5 and day 10 of endothelial infection, suggesting that surviving wild type USA300 between day 5 and day 10 may be potent persister SCVs.

### *S. aureus* can escape from infected cells

**Figure 5:**
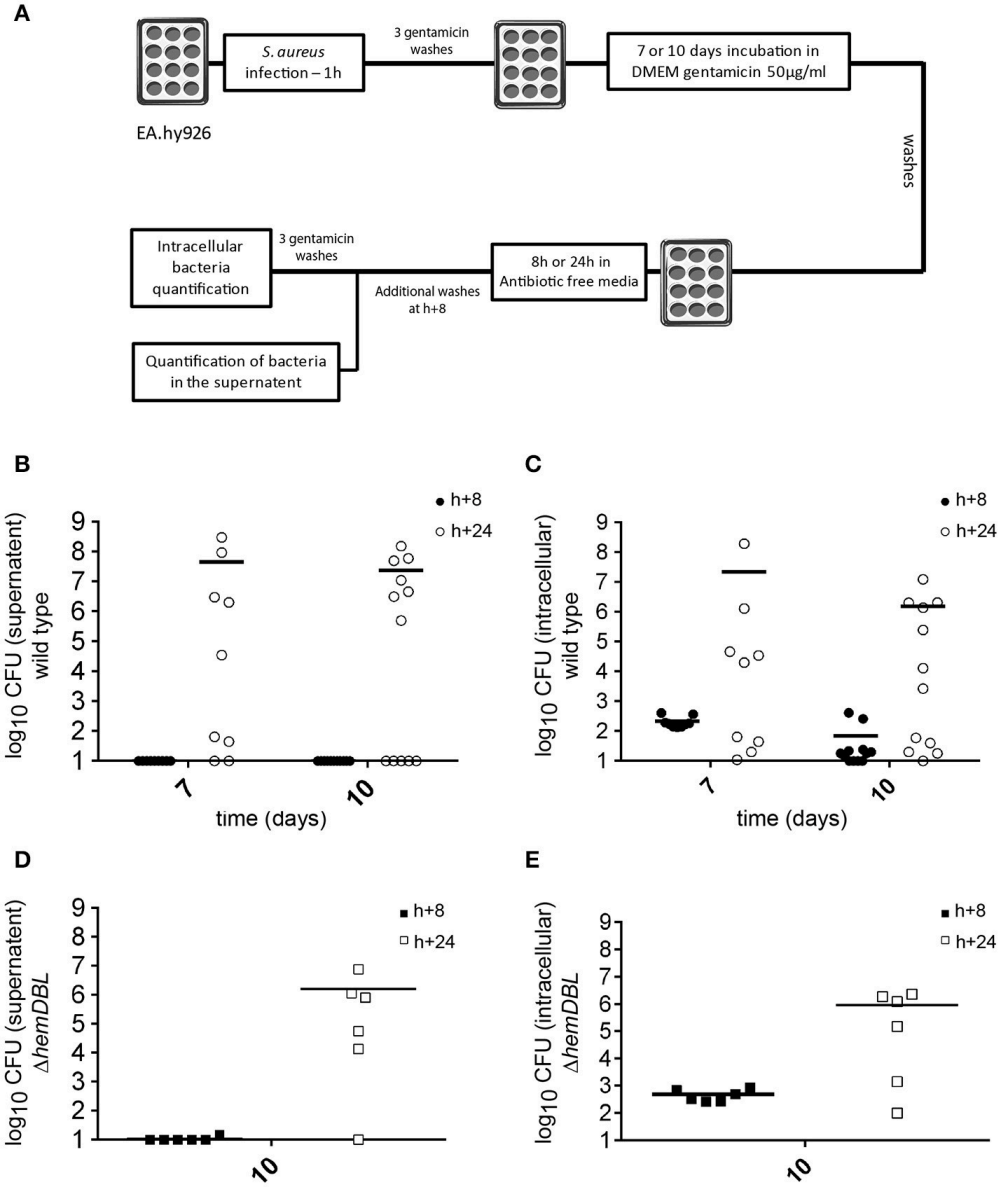
Intracellular *S. aureus* may escape from infected host cells up to 10 days after infection. **(A)** Schematic representation of the experimental set-up (see Materials and Methods). Briefly, EA.hy926 cells were infected as described. At day 7 and 10 after infection cell monolayer was washed 3 times with PBS containing gentamicin 300 μg/mL and 3 times with antibiotic free PBS. After 8 h of incubation in antibiotic free medium the number of bacteria in the supernatant was assessed. The cells are then incubated 16 h more hours in antibiotic free medium and bacteria load in the supernatant and inside cells was assessed. **(B–E)** Mean of viable bacterial load 8 h (black) and 24 h (white) after incubation with antibiotic free medium, 7 or 10 days after infection. **(B,D)** Bacterial load in the supernatant. **(C,E)** Bacterial load inside cells. **(B,C)** USA300-GFP *S. aureus*. **(D,E)** Δ*hemDBL* USA300-GFP. Ten CFU were considered as a detection threshold.

We next tested whether *S. aureus* USA300 could escape from infected EA.hy926 cells and re-seed the extracellular medium after prolonged intracellular persistence, upon removal of antibiotic treatment. For this, we set up the infectious scheme depicted in Figure 5A. Briefly, 7 and 10 days after infection, we removed gentamicin from the culture media (see Materials and Methods). Cells were then washed several times and incubated 8 h in antibiotic-free medium and supernatant were tested for the presence of *S. aureus*. No bacteria were detected in the cell culture media 8 h after removal of antibiotics, indicated that no detectable *S. aureus* survived in the extracellular media nor were released by cells early after removal of gentamicin. Cells were then incubated for 16 additional hours before counting extracellular and intracellular bacteria. At days 7 and 10, the number of *S. aureus* was dramatically increased in the supernatants of 5 out of 9 wells, and 7 out of 12 wells, respectively (Figures 5B,C). Bacterial multiplication in cell culture supernatant was associated with an increase in the number of intracellular CFUs of the corresponding well. Considering that we never observed an increase in the intracellular CFU count in the presence of gentamicin at day 7 and 10, these data tend to suggest that *S. aureus* USA300 that have been internalized for 7–10 days in endothelial cells are still able to evade the cells. However, as mentioned above we did not detect proliferation of intracellular wild type bacteria by time-lapse microscopy at day 7 (Video S6).

To confirm that single bacteria did not escape from the intracellular compartment by cell lysis (due to overwhelming intracellular proliferation as observed at day 1–3), we also tested the relapse of the Δ*hemDBL* mutant 10 days after infection (Figures 5D,E). As for the wild type parental strain, *S aureus* Δ*hemDBL* mutant bacteria proliferated in the supernatant to reach ≃10^6^ CFU in 5 wells out of 6. This proliferation was also associated with an increase in the intracellular CFU count.

Altogether, these data are compatible with the idea that single *S. aureus* USA300 bacteria are: (i) able evade endothelial cells after 10 days of intracellular persistence (either actively or passively, after cell extrusion or cell death), (ii) proliferate in the extracellular medium, and (iii) eventually infect neighboring cells, in the absence of antibiotics.

## Discussion

Having confirmed the increased intracellular persistence of *S. aureus USA300* in endothelial cells as compared to macrophages, we show here that the initial persistence of wild-type *S. aureus* in endothelial cells corresponds to distinct simultaneous situations at the single cell level. Indeed, time-lapse microscopy revealed that, although in most cases, rapid bacterial proliferation leading to cell lysis was observed; in some cells, a transient bacterial multiplication ultimately controlled by the host, as well as a prolonged bacterial persistence without apparent morphological cellular damage, could also be observed. This dynamic view of *S. aureus* intracellular persistence provides a complementary and necessary assessment of the intracellular lifestyle of the bacterium that should contribute to better understand the *in vivo* situation than any classical snapshot analysis.

### Persistence in endothelial cells corresponds to different single cell events

Two main outcomes of host cell invasion by *S. aureus* are generally described: (i) rapid host cell lysis, triggered by the secretion of toxins and other pro-inflammatory factors, which induce strong inflammatory and cytotoxic effects; (ii) persistence within morphologically-intact host cells for extended periods of time, where secretion of virulence factors is either down-regulated or not expressed (Proctor et al., 2014). Of note, these persisting bacteria generally correspond to so-called “small colony variants” (SCV). The association between *S. aureus* SCVs and persistent infection was first reported by Proctor and co-workers in 1995 (Proctor et al., 1995) and recent studies have shown that the impaired growth of *S. aureus* SCVs was due to decreased metabolic activity (Amato et al., 2014). Since a number of other pathogenic bacteria species have been shown to persist within their host as SCVs (Kahl et al., 2016), it may be considered as a major survival strategy in chronic bacterial infections. Summarizing the prolific literature addressing *S. aureus* intracellular persistence is quite complicated and may even be misleading. Indeed the comparison of results obtained in one experimental setup with those obtained in another one is quite often impossible. Of interest, recent studies have revealed that the general stress transcription Sigma B (SigB) was a major player of the dynamic switch between full virulence and long-term persistent phenotypes in wild-type strains. SigB by down-regulating the quorum sensing *agr* system, silences toxins production and improves persistence within the host cell (Tuchscherr et al., 2015).

Here, we thought to use video microscopy to follow the behavior of individual *S. aureus* USA300-GFP infected endothelial cells over a seven day-period. This technique has been very recently used by Jubrail et al. (2016) to visualize the fate of GFP-labeled *S. aureus* strain Newman in infected THP-1 macrophages, over a 72 h-period. This experiment showed that intracellular bacteria re-emerged from macrophage from 24 to 72 h. This leads to a constant reseeding of the extracellular compartment. Our experiment in endothelial cells revealed that, whereas most infected cells were unable to restrict bacterial growth and were lysed within the first 48 h, some cells were able to resolve the infection. In these cells, morphologically intact bacteria were observed at least for 7 days.

Thus, upon entry of *S. aureus* into endothelial cells, simultaneous single cellular fates coexist which could not have been anticipated from classical confocal microscopy analyses or quantification of viable intracellular bacteria at fixed time points.

### Persistence and exit of *S. aureus* from human cells

Our results and those of others indicate that, within the first hours after internalization, bacteria tend to actively multiply inside human cells (and ultimately lyse them) whereas infected cells attempt to restrict their proliferation. This selective pressure on *S. aureus* may favor the emergence of transient persisters which are able to survive into host cells for several days, without multiplication, before re-emerging in the extracellular compartment where they can rapidly proliferate and infect neighboring cells.

Of note, after several days of intracellular persistence, *S. aureus* is no longer capable of proliferation. Thus, the evasion of SCVs in the supernatant of infected cells (Figure 5D) in the absence of antibiotic, suggests that single non-proliferative bacteria are able to evade cells by an unknown, possibly active, process yet to be determined.

## Author contributions

GR, XT, FT, and MD performed the experiments; GR, AC, and MC designed the research; GR, XN, AC, and MC discussed the data and the manuscript; GR, AC, and MC co-wrote the manuscript.

### Conflict of interest statement

The authors declare that the research was conducted in the absence of any commercial or financial relationships that could be construed as a potential conflict of interest.
