# Supplementary material for: Intracellular Survival of Staphylococcus aureus in Endothelial Cells: A Matter of Growth or Persistence
Source: Front Microbiol. 2017 Jul 19;8:1354. doi: 10.3389/fmicb.2017.01354 (PMC5515828; doi:10.3389/fmicb.2017.01354)
Supplement: Supplementary file 7 [file DataSheet1.DOCX]

**Intracellular survival of *Staphylococcus aureus* in endothelial cells: a matter of growth or persistence**

Guillaume Rollin^1,2 ǂ^ , Xin Tan^1,2 ǂ^, Fabiola Tros^1,2^, Marion Dupuis^1,2^, Xavier Nassif^1,2,3^, Alain Charbit^1,2,^ * and Mathieu Coureuil^1,2,^ *

**Video S1. Time lapse microscopy of cells following USA300-GFP internalization in EA-hy296 cells.** USA300-GFP: green; DNA of dying cells and permeable stained with propidium iodide: red. Time is in hours. Bar = 50µm.

**Video S2.** Crop of Video S1 showing bacteria growth inside Ea.Hy926 cells, which leads to cell lysis and realease of bacteria.

**Video S3.** Crop of Video S1 that shows bacteria growth inside Ea.Hy926 cells, which leads to cell death.

**Video S4.** Crop of Video S1 that shows host cell control and elimination of bacteria.

**Video S5. Time lapse microscopy of cells following Δ*hemDBL* USA300-GFP mutant internalization in EA-hy296 cells.** USA300-GFP: green; Time is in hours. Bar = 50µm.

**Video S6. Time lapse microscopy of cells, 7 days after USA300-GFP internalization in EA-hy296 cells.** USA300-GFP: green; Time is in hours. Bar = 50µm.
